# Serum variations of anti-mullerian hormone and total testosterone with aging in healthy adult Iranian men: A population-based study

**DOI:** 10.1371/journal.pone.0179634

**Published:** 2017-07-17

**Authors:** Fahimeh Ramezani Tehrani, Mohammad Ali Mansournia, Masoud Solaymani-Dodaran, Sonia Minooee, Fereidoun Azizi

**Affiliations:** 1 Reproductive Endocrinology Research Center, Research Institute for Endocrine Sciences, Shahid Beheshti University of Medical Sciences, Tehran, Iran; 2 Department of Epidemiology and Biostatistics, School of Public Health, Tehran University of Medical Sciences, Tehran, Iran; 3 Minimally Invasive Surgery Research Center, Iran University of Medical Sciences, Tehran, Iran; 4 Reproductive Endocrinology Research Center, Research Institute for Endocrine Sciences, Shahid Beheshti University of Medical Sciences, Tehran, Iran; 5 Endocrine Research Center, Research Institute for Endocrine Sciences, Shahid Beheshti University of Medical Sciences, Tehran, Iran; Universite Clermont Auvergne, FRANCE

## Abstract

**Background:**

Literature proves anti-mullerian hormone (AMH) and total testosterone (TT) as two important reproductive hormones in male development, however evidence regarding age variations of these hormones is lacking.

**Aims:**

To estimate the normal serum AMH values and to assess the age-specific TT levels in men aged 30–70, we conducted the present population-based study.

**Methods:**

A total of 831 healthy eligible men, aged 30–70 years, were recruited from Tehran Lipid and Glucose study cohort. Centiles for AMH were estimated according to the exponential normal 3-parameter model. The parametric method of Royston available in general software was applied for the first time to estimate the age-specific AMH and TT percentiles of 5^th^, 10^th^, 25^th^, 50^th^, 75^th^, 90^th^ and 95^th^.

**Results:**

Mean AMH level was 6.93, ranging from 0.1 to 40.1 ng/ml. Serum AMH concentrations followed a steady reduction with increasing age. Mean TT level was 4.8, ranging from 0.44 to 11.4 ng/ml.

**Discussion:**

A measurable serum concentrations of AMH in healthy males throughout lifespan with variations, based on age, confirming a slight age-related AMH decline. Fractional polynomial (FP) regression models revealed that the mean and standard deviation (SD) of the TT were not associated with age, so the percentiles estimated were not age-specific.

**Conclusion:**

We presented a nomogram of age-specific AMH values in a healthy cohort of Iranian men. This finding might have clinical importance in dealing hormonal disorders in men.

## Introduction

The age-specific variations for hormones particularly sex hormone levels throughout life has drawn interest in recent years. Anti-Mullerian Hormone (AMH) is a dimeric glycoprotein and a member of the transforming growth factor β (TGF- β) family of growth factors [1]. It is secreted by the Sertoli cells surrounding the genocytes and has the main role in male sexual differentiations [2].

In clinical pediatric endocrinology the role of AMH, as a marker of Sertoli cells function, in addition to the testosterone levels, as a marker of Ledig cells function, have been well established[3]. Specifically, the role of AMH in the diagnosis of boy's hypogonadism[4], anorchia[5], cryptorchidism[6] and disorders of sex development[7]has been previously confirmed. Besides evaluating the gonadal function, there exist reports indicating AMH as a predictor of renal[8] or cardiovascular diseases in middle-aged or older men[9]; suggesting new insights from a clinical standpoint. However, the pattern of AMH secretion varies greatly among different age groups and also between the elderly men of similar age [10]. Therefore generating and facilitating a nomogram for age-specific serum AMH levels in men might help clinicians to better elaborate the related medical conditions.

Testosterone as an AMH regulator has the potential of decreasing AMH expression in Sertoli cells. The evidence is based on the inverse association between testosterone and AMH levels in the development process of males[11]. As the Sertoli cells undergo their maturation, the androgen-dependent down-regulation of AMH[12, 13] and the inhibitory effect of androgens on AMH production in pubertal boys occur[14]. However, due to the lack of androgen receptors expression, this negative association is not observed during early fetal or neonatal periods[15]. Despite the studies showing the AMH-testosterone interactions during early human development or pubertal periods, few have been conducted to seek the AMH and testosterone variations simultaneously among middle-aged or older men[10, 16].

Testosterone undergoes age-related production changes that are considered as a normal process in male aging [17], however, the trend of total testosterone (TT) variations with advancing age is slightly debatable among different papers. A number of cross-sectional and longitudinal observations have demonstrated decreasing TT with age [18–20]; as for example, in the prospective cohort study by Travison et al., a substantial age-independent reduction in TT levels of 2769 American men aged 49-75was reported[21]. In contrast, some other studies stated no change or even found an increase in TT values in middle-aged men [22], in particular, the prospective cohort study of Health in Men, among 4263 men aged 70–89 years, reported a relative stable serum level of TT[23].

To date, although many studies have evaluated AMH concentrations in men [24–26]; of them, few have examined trend of AMH changes in elderly[10, 16]. In a relevant population-based study, Aksglaede et al. described the ontogeny of AMH secretion among 1027 Danish healthy male infants, children, adolescents and adult males from birth to the age of 69. They found a relative stable level of AMH throughout adulthood; however, the findings may mostly represent the normal range of AMH in boys from infancy to the age of 19; since in that study 115 participants were adults, aged 20–69 years[16].

Almost all previous nomograms presented for TT and AMH values were separate from each other and were not done as a single study. We aimed to integrate these values in a cohort of adult middle-aged and older men. The subjects were extracted from a population-based cohort of healthy Iranian men [27]. Also, for the first time, the parametric method of Royston was recruited to estimate the age-specific percentiles[28].

## Materials and methods

This is a cross sectional study conducted as a part of the TLGS (Tehran Lipid and Glucose Study) cohort phase 1, an ongoing community based prospective study initiated in 1999[27]. A total of 831 eligible healthy men, aged 30–70 year, were extracted from TLGS cohort; participants with incomplete data, unavailable blood samples, history of chronic disease, testicular or endocrine disorders and use of certain medications (such as antiandrogens, gonadotropin-releasing hormone agonists, glucocorticoids, 5-alfa reductase inhibitors, opiates, antiepileptic and hormone therapy) were excluded. Subjects were residence of district No.13 of Tehran and were under the coverage of three medical health centers, selected by multistage cluster random sampling method.

A standardized questionnaire covering demographic data, smoking and physical activity habits and medical and familial history was completed. Initially the participants were interviewed and then referred to laboratory for blood sampling. The study proposal was approved by the Medical Ethics Committee of the Research Institute for Endocrine Sciences and informed consent was obtained from the participants.

Details of physical examinations, anthropometric and lab measurements which were performed by trained examiners have been previously published[29]. Following phlebotomy, blood samples were drawn between 07:00 and 09:00 a.m. after an overnight fasting condition and then centrifuged within 30–45 min of collection and stored at -80°C.

Determination of TT was done based on Enzyme Immunoassay (EIA) (DRG Instrument, Sunrise, Tecan Co. Salzburg, Austria, GmbH, Germany). The intra-interassay coefficients of variations (CVs) were 5.7% and 8.4% respectively at the detection limit of 0.002 ng/ml. Follicle stimulating hormone (FSH), luteinizing hormone (LH) and prolactin (PRL) values were measured using Immunoradiometric assay (IRMA) (Izotop, Budapest, Hungary, Gamma couter: Dream Gamma- 10, Goyang-si, Gyeonggi-do, South Korea) with the intera-interassay coefficients of variations (CVs) of 1.3–1.4%, 2.9–3.0% and 2.5–2.6%, respectively, at the detection limit of 0.08 mIU/mL, 0.02 mIU/mL and 0.04 ng/mL.

Total AMH was determined using EIA (AMH Gen Π, Beckman Coulter, Inc. Ca, USA, Sunrise, Tecan Co. Sazburg, Austria). The modified protocol was used for measuring AMH, as the original Gen Π may underestimate serum AMH levels[30]. The intra-interassay CVswere3.1% and 3.2% respectively at the detection limit of 0.08ng/ml. All analyses were performed at the TLGS research laboratory.

### Statistics

Baseline characteristics are expressed as mean± standard deviation (SD) and frequency± percentage. Normality of distribution was examined by Kolmogorov-Smirnov test. The normal-based methodology described by Altman and Chitty[31], Royston and Wright[28, 32]and Tehrani et al [33]was used to estimate age-specific AMH percentiles. Fractional polynomial (FP) regression models were fitted separately to estimate the mean and SD of the log AMH values as functions of age; SD was modeled using the scaled absolute residuals from the estimated regression model for the mean. Based on a closed-test comparison between FP models, regression models linear in age were selected for both mean and SD. Percentiles were obtained by combining these two regression models, using the assumption that the conditional distribution of log AMH values given age is normal. Percentile curves on the original scales (AMH nomogram) were obtained by taking antilogs of the calculated curves.

The normal plot of the Z-scores from the normal model described above showed that normality does not hold. An exponential–normal (EN) 3-parameter model[28] provided better fit with a deviance significantly lower than the simpler normal model (p<0.001), and the normal plots of the Z-scores from the EN model appear reasonably linear. The EN model was fitted by maximum likelihood using the Stata command xriml[34].

**Fig 1:**
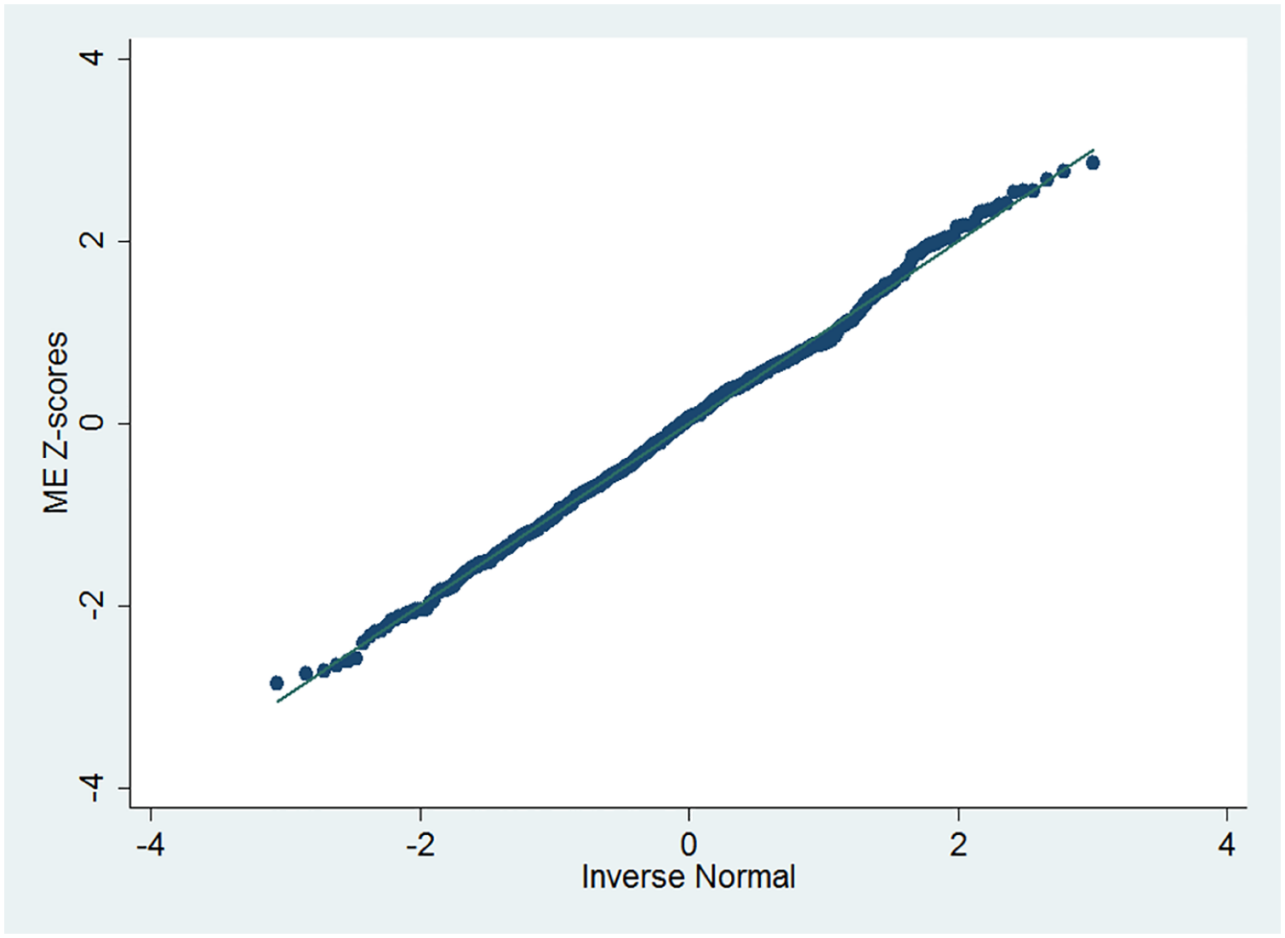
Normal Q-Q plot of the Z-scores from the modulus exponential–normal (MEN) model for estimating testosterone percentiles.

Fractional polynomial (FP) regression models revealed that the mean and standard deviation (SD) of the testosterone were not associated with age, so the percentiles estimated were not age-specific. A modulus exponential–normal (MEN) 4-parameter model[28]provided better fit with a deviance significantly lower than the simpler normal model (p<0.001) or exponential–normal (EN) 3-parameter model (p<0.001), and the normal plots of the Z-scores from the MEN model appear reasonably linear (Fig 1), (P-value for the Shapiro-Wilk test = 0.12). The 2.5^th^, 5^th^, 10^th^, 25^th^, 50^th^, 75^th^, 90^th^, 95^th^ and 97.5^th^ percentiles of testosterone levels were calculated. The MEN model was fitted by maximum likelihood using the Stata command xriml[34].

## Results

Characteristics of study participants are represented in Table 1. Mean age and BMI of the men were 46.2 years (±0.4) and 25.9 kg/m^2^(±0.14), respectively. Mean serum AMH level in healthy men, aged 30–70 years, was 6.93, ranging from 0.1 to 40.1ng/ml., while lowest values for AMH were detected in the 61–70 year age group. The mean serum TT level in healthy men aged 30–70 years was 4.8, ranging from 0.44 to 11.4 ng/ml. The lowest values for testosterone were detected in the age group of 51–60 years.

**Table 1:** Characteristics of the study participants* (n = 831).

Age (years)	46.3(11.)
Body mass index (kg/m^2^)	25.9(4)
Waist circumference (cm)	88.9(11)
Hip circumference (cm)	96.3(7.2)
Systolic blood pressure (mmHg)	121.2(19.8)
Diastolic blood pressure (mmHg)	78.6(11.1)
Fasting blood sugar (mg/dl)	99.3(31.6)
Triglycerides (mg/dl)	180.7(102.2)
Total cholesterol (mg/dl)	208.7(40.2)
High-density lipoprotein (HDL-c) (mg/dl)	38.7(9.4)
Low-density lipoprotein (LDL-c) (mg/dl)	134.4(34.2)
Luteinizing hormone (mIU/mL)	4.6(3.1)
Follicle stimulating hormone (mIU/mL)	6.7(5.5)
Prolactin (ng/ml)	9(13.3)
Daily smoker; No.(%)	627(25.2%)
Smoking < 10 cig/day; No.(%) Smoking > 10 cig/day; No.(%)	345(13.9%) 300(12%)
AMH (ng/ml)	6.9(5)
AMH in 30–40 years; No.(%) = 337(40.55%) AMH in 41–50 years; No.(%) = 175(21%) AMH in 51–60 years; No.(%) = 164(19.73%) AMH in 61–70 years; No.(%) = 155(18.65%)	8(5.6) 6.5(4.2) 6.4(4.7) 5.3(4.3)
TT (ng/ml)	4.8(1.5)
TT in 30–40 years; No.(%) = 337 (40.55%) TT in 41–50 years; No.(%) = 175 (21%) TT in 51–60 years; No.(%) = 164 (19.73%) TT in 61–70 years; No.(%) = 155 (18.65%)	4.8(1.4) 4.8(1.5) 4.6(1.2) 4.9(1.7)

AMH, Anti- Mullerian hormone; TT, total testosterone

*Mean(SD), except where otherwise indicated.

**Fig 2:**
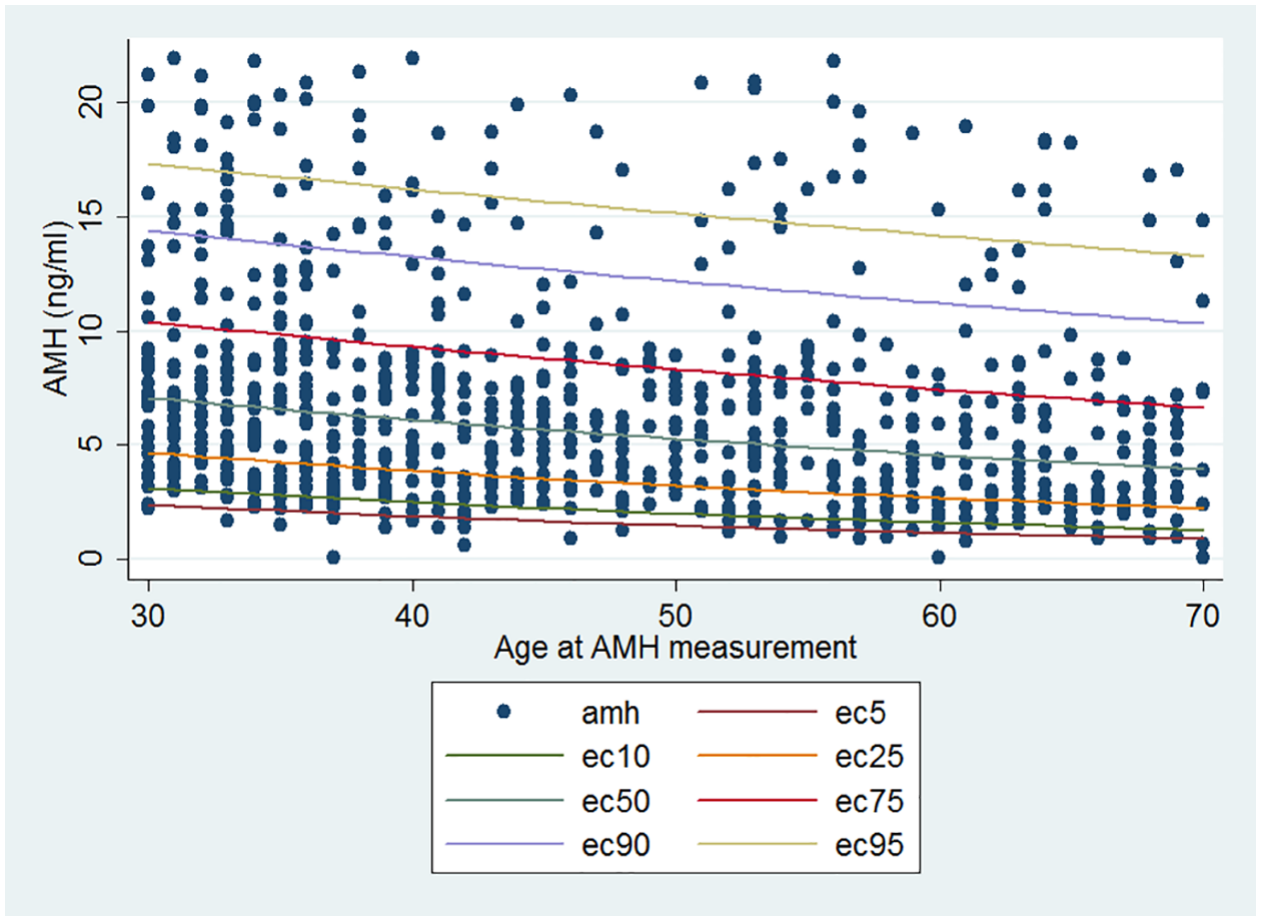
Anti-mullerian hormone percentiles in healthy men aged 30–70 years. AMH = anti-mullerian hormone (the lines indicate 5th, 10th, 25th, 50th, 75th, 90th and 95th percentiles of AMH levels).

Fig 2 displays AMH levels and the estimated values for age-specific 5^th^, 10^th^, 25^th^, 50^th^, 75^th^, 90^th^ and 95^th^ centiles in 831 participants; indicating a gradual decline of AMH values in adult males. A summarization of the age-specific AMH decrease and the corresponding percentiles is presented in Table 2.

**Table 2:** Normative age-specific anti- mullerian hormone reference values and corresponding percentiles in men.

Percentiles							
Age (years)	5^th^	10^th^	25^th^	50^th^	75^th^	90^th^	95^th^
30	2.41	3.12	4.68	7.08	10.40	14.39	17.31
31	2.36	3.06	4.59	6.98	10.29	14.27	17.19
32	2.30	2.99	4.51	6.88	10.17	14.15	17.08
33	2.25	2.92	4.43	6.78	10.06	14.03	16.97
34	2.19	2.86	4.35	6.68	9.95	13.92	16.85
35	2.14	2.80	4.27	6.58	9.84	13.80	16.74
36	2.09	2.74	4.19	6.49	9.73	13.69	16.63
37	2.04	2.68	4.11	6.39	9.62	13.57	16.52
38	1.99	2.62	4.04	6.30	9.52	13.46	16.41
39	1.94	2.57	3.97	6.21	9.41	13.35	16.30
40	1.90	2.51	3.89	6.12	9.30	13.24	16.19
41	1.85	2.46	3.82	6.03	9.20	13.13	16.09
42	1.81	2.40	3.75	5.94	9.10	13.02	15.98
43	1.76	2.35	3.69	5.86	9.00	12.92	15.87
44	1.72	2.30	3.62	5.77	8.90	12.81	15.77
45	1.68	2.25	3.55	5.69	8.80	12.70	15.66
46	1.64	2.20	3.49	5.61	8.70	12.60	15.56
47	1.60	2.15	3.43	5.53	8.60	12.49	15.46
48	1.56	2.11	3.36	5.45	8.51	12.39	15.35
49	1.53	2.06	3.30	5.37	8.41	12.29	15.25
50	1.49	2.02	3.24	5.29	8.32	12.19	15.15
51	1.45	1.97	3.19	5.21	8.23	12.09	15.05
52	1.42	1.93	3.13	5.14	8.14	11.99	14.95
53	1.39	1.89	3.07	5.06	8.05	11.89	14.85
54	1.35	1.85	3.02	4.99	7.96	11.79	14.75
55	1.32	1.81	2.96	4.92	7.87	11.69	14.65
56	1.29	1.77	2.91	4.85	7.78	11.59	14.56
57	1.26	1.73	2.85	4.78	7.69	11.50	14.46
58	1.23	1.69	2.80	4.71	7.61	11.40	14.36
59	1.20	1.66	2.75	4.64	7.52	11.31	14.27
60	1.17	1.62	2.70	4.57	7.44	11.22	14.17
61	1.14	1.59	2.65	4.50	7.36	11.12	14.08
62	1.12	1.55	2.61	4.44	7.28	11.03	13.99
63	1.09	1.52	2.56	4.37	7.20	10.94	13.89
64	1.06	1.49	2.51	4.31	7.12	10.85	13.80
65	1.04	1.45	2.47	4.25	7.04	10.76	13.71
66	1.01	1.42	2.42	4.19	6.96	10.67	13.62
67	.99	1.39	2.38	4.13	6.88	10.58	13.53
68	.97	1.36	2.33	4.07	6.80	10.50	13.44
69	.94	1.33	2.29	4.01	6.73	10.41	13.35
70	.92	1.30	2.25	3.95	6.65	10.32	13.26

**Fig 3:**
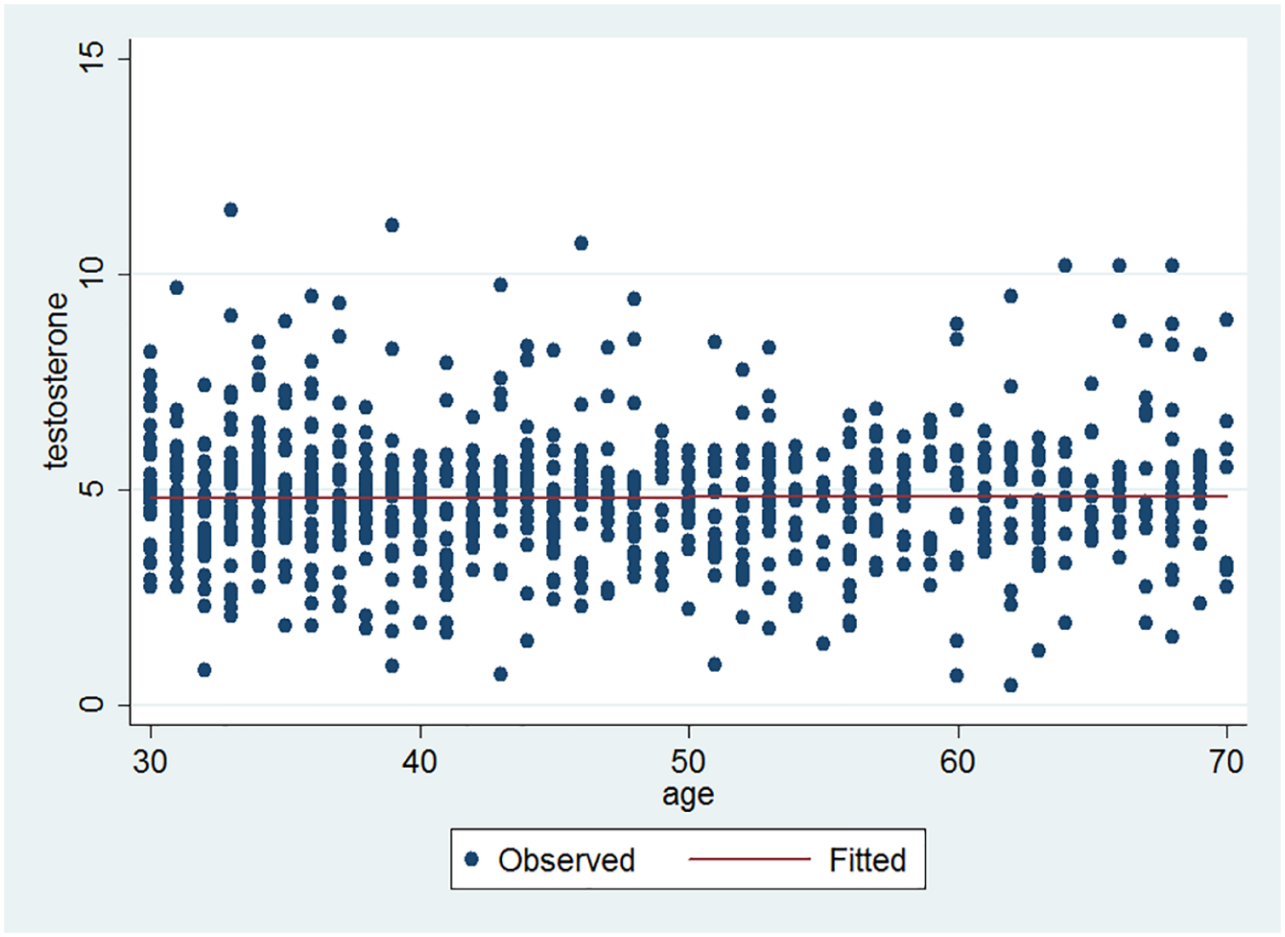
Scatterplot of testosterone versus age and fitted regression line (Pearson r = 0.016; P-value = 0.65).

The 2.5^th^, 5^th^, 10^th^, 25^th^, 50^th^, 75^th^, 90^th^, 95^th^ and 97.5^th^ percentiles of testosterone levels were as following; 2.04, 2.57, 3.13, 3.99, 4.77, 5.60, 6.66, 7.46 and 8.28. Fig 3 depicts a stable level of serum TT with no increase or decrease with advancing age throughout lifespan.

Using bivariate correlation, significant inverse relation between AMH and age (r = -.19, p< 0.001) and BMI (r = -0.08, p = 0.01) was shown. Partial correlation analysis between AMH, lipid and hormonal factors, after controlling for age and BMI, indicated that except for FSH and LH that were inversely correlated with AMH (r = -0.15, p<0.001 and r = -0.08, p = 0.01, respectively), there was no significant relation between other parameters.

## Discussion

In our study, we found measurable serum concentrations of AMH in healthy males throughout lifespan with variations, based on age, confirming a slight age-related AMH decline.

A variety of nomograms have been created for fertile or infertile women so far[35–37], but the age-specific AMH levels in men using the mentioned statistical approach, has to the best of our knowledge, not been studied before. There is only one report on the ontogeny of AMH secretion in healthy males which was mostly focused on the serum values from infancy until puberty periods [16]. The only related available study was conducted among 1027 Danish healthy male infants, children, adolescents and adult males from birth to the age of 69. Participants were recruited from three population-based studies [38–40]; however, since in that study 115 participants were adults, aged 20–69 years and the rest were boys from infancy to the age of 19, the results may not completely represent the normal range and fluctuations of AMH in adult men. Therefore, we believe that our study might be confirmatory in terms of adulthood age ranges.

Consistent with previous findings in elderly men[10, 16], we observed a gradual reduction in AMH values with advancing age which may be due to the age-related decrease of Sertoli cells [41]. After controlling for age and BMI factors, the serum AMH levels had only negative significant correlation with FSH and LH; suggesting age-related reduced testicular function, that is in agreement with previous reports both in male [42] and female populations [43].

Dennis et al introduced AMH as a novel regulator of the cardiovascular system and declared that serum AMH levels in healthy older men inversely correlate with the diameter of their abdominal aorta [9]. In this regard, related animal studies on AMH association with atherosclerosis are available for rhesus monkeys [44].

In a recent study by Qayyum and Akbar[45], an independent and inverse relation between serum AMH and all-cause mortality in men was revealed. In this 9-year follow-up of 989 American adult men, they found that those who were alive at the end of the study had higher levels of AMH than those who died within follow-ups. It may indicate potential effects of AMH in relation to mortality risk factors; however, the study lacks adequate sample size and multiple measurements of AMH during follow-ups.

Given these data, the applicability of AMH needs to be further investigated. Also, sparse publications regarding AMH practical implications in men necessitate the presentation of a nomogram for AMH based on age percentiles. An applicable implication of our observations is that AMH nomograms in men might provide information regarding different health statuses. Although, data is limited in this regard, recent evidence states that AMH is no longer a mere reproductive hormone and perhaps has regulatory effects in diseases like lung cancer[46] or as above mentioned in cardiovascular disorders [9].

There are several statistical methods for estimating age-specific percentiles and reference intervals. However, the following three methods are the most widely applied approaches in practice: HRY method of Healy et al[47], LMS method of Cole[48], and normal methodology of Royston[28]. The parametric method of Royston used in the present study, is a simple method available in general software whose validity is comparable with the other two methods and provides simple formula for estimation of an individual's centile and allows formal statistical inference for comparing models using likelihood ratio tests.

Also, we found no change in TT concentrations with advancing age. Our observations are in line with some previous findings. Using model selection and validation data analysis extracted from thirteen studies, Kelsey et al. validated a normative model for TT throughout lifespan and in average found no evidence of TT fall in men over 40 years and hence did not support the andropause phenomenon [49]. This model had the strength of recruiting TT measurements of over 10,000 healthy men aged 3–101 years.

Similarly, the study of Yeap et al among 3645 men aged 70–89, proved no testosterone decline within aging [23]. Frost et al reported[50] the same reference intervals for testosterone among healthy young (11.7–37.7 nmol/l, aged 20–29) and their elderly counterparts (11.2–37.8 nmol/l, aged 60–74) was reported.

Comparatively, there exists contradictory data. The longitudinal studies of Baltimore aging [18], Massachusetts male aging study [51] and population-based study of Tromso[52], all reported an annual modest decline of approximate 0.3–0.5% in serum TT levels. This discordance between present paper and the mentioned studies may be attributed to the age range recruited, cross-sectional or longitudinal characteristic of the studies and accompanied influencing factors like obesity or smoking habits.

Interestingly, Halmenschlager et al (n = 428)[22]and Rhoden et al. (n = 1,071)[53]observed not only no decrease in TT levels in variance but stated increase with aging. However, in general, a majority of studies concluded no change or a slight decline in TT levels of aging men. Unfortunately, few studies are dedicated to the men older than age 65 years, while a large number of undesirable symptoms of androgen deficiency occur later in life.

Taken altogether, we observed a gradual decline in AMH levels with aging but we found no significant reduction in testosterone levels. Perhaps, the underlying fact might be that, as previous studies have agreed [54, 55], the levels of hormones secreted by testes are independent and they do not necessarily adjust each other.

It is the first population based survey on healthy Iranian men (aged 30–70) to access the trend of AMH and testosterone changes. Strength of this study is its population-based design and the statistical approach in estimating hormones percentiles. However, our study is subject to limitations. In this study the available AMH measurement kit was the standard method of AMH Gen II assay which was the main AMH measurement method in the past few years, but may be less superior to some other newly introduced immunoassay methods in terms of sample dilution and sample storage[56]. However, not all the studies have challenged this pitfall and some that have compared different AMH measurement methods, have found good correlations between AMH values measured by Gen II assay and other new assay methods[57].

Also, due to the laborious and costly reference laboratory method which requires equilibrium dialysis, we were not able to measure free testosterone. Our primary aim was to assess TT variations in elderly; however, this limitation may be explained by the results of the studies which clearly support the stronger association of total testosterone with some androgenic disorders in men compared with free hormone[58]. We were not able to measure sex hormone binding globulin (SHBG)levels which could better interpret the hormone variations over time; however, by applying a strict inclusion criteria and by excluding the subjects with different health problems, we attempted to select our study sample from a cohort of healthy men to avoid the effect of different diseases on increasing or decreasing SHBG dosage which could contribute to incorrect total hormone estimations.

Also, we assessed the men aged 30–70 years, so the findings do not comprise the age groups below or above the designated age ranges studied here; hence for the generalization of our results, further extensive epidemiologic studies among different racial groups are needed.

In conclusion, we observed a slight decrease in AMH values and stable concentrations of testosterone in middle-aged and older men with aging. Accessing to the normal range values of AMH and testosterone may have the potentiality of evaluating or predicting specific health conditions.
